# Either Sent by God or Used by God: Impact of COVID-19 on the Religious Lives of Black Families With Dementia

**DOI:** 10.1093/geroni/igab046.1788

**Published:** 2021-12-17

**Authors:** Fayron Epps, Yiran Ge, Mayra Sainz, Janelle Gore

**Affiliations:** Emory University, Atlanta, Georgia, United States

## Abstract

The COVID-19 pandemic has underscored systemic disparities and laid its effects on the Black community. Often overlooked is how health disparities heighten stress and affect the emotional well-being of Black American caregivers. The purpose of this study is to explore the impact of COVID-19 on church engagement for Black families affected by dementia. A qualitative design was employed to collect data from current caregivers, faith/church leaders, and persons with cognitive impairment. Participants (n = 17) were predominantly female, all identified as Black. During semi-structured interviews, participants were asked how COVID-19 has impacted their participation in faith practices. The following themes emerged: (a) ability to continue faith practices, (b) increased church engagement, (c) new normal, (d) importance of fellowship, and (e) role of technology. Participants believed COVID-19 did not impact their faith practice partly due to the ability to continue with faith traditions in a virtual format. Online worship services enabled more families affected by dementia to participate. Many church leaders expressed the intent of continuing to provide online worship services post-pandemic. Families highlighted their need to have fellowship with other parishioners. Technology was perceived as a double-edged sword that serves as both a motivator and a barrier to religious engagement. These findings will support faith leaders and churches in understanding the needs of their congregation during the COVID-19 pandemic, specifically, it will allow families living with dementia to continue engaging in religious activity and living in meaningful ways.

